# Effectiveness of a training intervention to improve the management of vertigo in primary care: a multicentre cluster-randomised trial, VERTAP

**DOI:** 10.1186/s13063-022-06548-7

**Published:** 2022-07-29

**Authors:** Jenniffer Elizabeth Pérez Patiño, José Lluís Ballvé Moreno, Yolanda Rando Matos, Jesús Almeda Ortega, Oriol Cunillera Puértolas, Ricard Carrillo Muñoz, Iván Villar Balboa, Xavier González Compta, Olga Lucía Arias Agudelo, Sebastiá Calero Muñoz, Vanessa Monforte Rodríguez, Anna Navarro Cortes, Eva Peguero Rodríguez

**Affiliations:** 1 Primary Care Centre Sant Martí de Provençals, Management Area of Barcelona, Catalan Institute of Health, Barcelona, Spain; 2 Vertigo Approach Research Group in Primary Care (VERTAP), Fundació Institut Universitari per la Recerca en Atenció Primària de Salut Jordi Gol i Gurina (IDIAPJGol), Barcelona, Spain; 3 Primary Care Centre Florida Nord, Management Area Metropolitana Sud, Catalan Institute of Health, Barcelona, Spain; 4 University of Barcelona, Barcelona, Spain; 5 Research Support Unit Metropolitana Sud, Institut Universitari d’Investigació en Atenció Primària Jordi Gol (IDIAP Jordi Gol), Cornellà de Llobregat, Spain; 6 Primary Care Centre Florida Sud. Management Area Metropolitana Sud, Catalan Institute of Health, Barcelona, Spain; 7 Ear, Nose and Throat Department, University Hospital Bellvitge, Barcelona, Spain; 8 Management Area Metropolitana Sud, Catalan Institute of Health, Barcelona, Spain; 9 Primary Care Centre, Management Area Metropolitana Sud, Catalan Institute of Health, Barcelona, Spain; 10 Rehabilitation Centre Viladecans, Management Area Metropolitana Sud, Catalan Institute of Health, Barcelona, Spain; 11 Primary Care Centre Castelldefels, Management Area Metropolitana Sud, Catalan Institute of Health, Barcelona, Spain

**Keywords:** Benign paroxysmal positional vertigo, Primary care, Randomised clinical trial, Training intervention

## Abstract

**Background:**

Benign paroxysmal positional vertigo (BPPV) is the most common type of vertigo. While BPPV is best treated with canalicular repositioning manoeuvres, they are not routinely performed in primary care (PC).

**Methods:**

To evaluate the effectiveness of blended training (online and face-to-face) on the diagnosis and management of vertigo to improve adherence of family doctors to clinical practice guidelines, we designed a community multicentre cluster-randomised open-label trial with an intervention (IG) and a control (GC) group of 10 primary care teams (PCT) each. Outcome variables will be ICD-10 diagnostic codes (proportion of nonspecific diagnoses such as dizziness and vertigo versus specific diagnoses such as BPPV, vestibular neuritis, and Menière’s disease); number of referrals to ENT or neurology specialists; prescription of antivertigo agents; and duration of sick leave due to vertigo. The baseline comparability of the two study groups will be analysed to ensure homogeneity. A description of all baseline variables will be performed. Student’s *t*-test will be used to evaluate the differences between the groups. Logistic regression multivariate analysis will be performed to study the relationship between baseline variables of professionals and centres with outcome variables.

**Discussion:**

With the improvement of the diagnosis and management of vertigo by family doctors after this training, we expect an increase in the proportion of specific diagnoses, a decrease in the prescription of antivertigo agents, a decrease in referrals to ENT or neurology specialists and a reduction in the duration of sick leave due to temporary disability. The blended training will be easily expanded within primary care services, since it is mainly delivered online, with a single face-to-face session to ensure that the manoeuvres have been adequately learned.

**Trial registration:**

ClinicalTrials.govNCT04929444. Registered June 18, 2021. This protocol has been approved by the Ethics Committee of the Institut Universitari d’Investigació en Atenció Primària Jordi Gol (IDIAP Jordi Gol) with the code 20/004-P. All patient data will be anonymised in agreement with the 2016/679 European Regulation.

**Supplementary Information:**

The online version contains supplementary material available at 10.1186/s13063-022-06548-7.

## Administrative information

Note: The numbers in curly brackets in this protocol refer to the SPIRIT checklist item numbers. The order of the items has been modified to group similar items (see http://www.equator-network.org/reporting-guidelines/spirit-2013-statement-defining-standard-protocol-items-for-clinical-trials/).

**Table:** 

Title {1}	Effectiveness of a training intervention to improve the management of vertigo in primary care: a multicentre cluster-randomised trial, VERTAP
Trial registration {2a and 2b}.	ClinicalTrials.gov Identifier: NCT04929444. Registered June 18, 2021, https://clinicaltrials.gov/ct2/show/NCT04929444
Protocol version {3}	Protocol version 1.0. June 18, 2021
Funding {4}	This study was awarded a Carlos III Health Institute Health Research Fund (FIS) competitive grant in 2019. The Carlos III Health Institute has not been involved in any part of the study.
Author details {5a}	JLBM conceived the study. JEPP, YRM, RCM, IVB, SCM, VMR, OLAA and EPR initiated the study design and collaborated in its implementation. XGC contributed his experience as an ENT specialist and ANC his experience in rehabilitation. This study is part of JEPP’s doctoral thesis. JAO and OCP contributed their statistical expertise in the design of the clinical trial. SIDIAP will perform the primary statistical analysis. The Catalan Society of Family and Community Medicine (CAMFiC) will collaborate with the implementation of the online course. All authors contributed to improving the study protocol and approved the final manuscript.
Name and contact information for the trial sponsor {5b}	Jordi Gol i Gurina Foundation **Address:** Gran Via de les Corts Catalanes, 587 Town 08007 Barcelona **Telephone:** 93 482 41 24 **Contact:** idiap@idiapjgol.org **Web address:** http://www.idiapjordigol.com/
Role of sponsor {5c}	The funding source had no role in the design of this study and will not have any role during its execution, analyses, interpretation of the data or decision to submit results.

## Introduction

### Background and rationale {6a}

Dizziness affects 20–30% of people at some point in their lives [1]. Approximately 5% of primary care consultations are due to dizziness, and over 50% of patients with dizziness are firstly assessed by family doctors [2, 3].

Vertigo, defined as the sensation of spinning objects and instability, severely affects the quality of life, causes a twofold increase in the prevalence of functional disability, worsens symptoms of depression [4] and decreases participation in social activities and self-efficacy to prevent falls [5]. A total of 69.8% of patients with vertigo have to reduce their work activities, and 63.3% need to take sick leave for several days [6]. Vertigo is associated with high costs for health services [6].

Benign paroxysmal positional vertigo (BPPV) is the most common cause of peripheral vertigo, affecting between 17 and 42% of patients with vertigo [7]. Up to 85–95% of cases of BPPV affect the posterior canal (BPPV-PC), and the Dix-Hallpike test (DHT) is considered the gold standard for its diagnosis [7]. As shown in literature reviews [8–11], the Epley manoeuvres (EM) are the most effective for treating BPPV-PC [12]. Consequently, with the use of the DHT and the EM, patients can be quickly identified and treated during primary care consultations without the need for referral or expensive medical tests. Clinical practice guidelines recommend the DHT and the EM and advise against the use of medical tests (except in the few cases where the diagnosis is unclear) and against the use of antivertigo agents [7].

Despite these recommendations, 60 to 80% of patients are visited in PC and hospital emergency departments where these diagnostic and treatment tests are not routinely performed [13–15]. A study by Dunlap et al. (2019) [16] based on 20.6 million PC visits (95% CI 17.3, 24.0) for dizziness observed a low percentage of treatments such as repositioning manoeuvres and a frequent diagnosis of “nonspecific dizziness” (75%).

Three main reasons were given for not implementing these diagnostic and therapeutic procedures (especially DHT and EM) in primary care: (1) the evidence originates from studies conducted in clinics with specialists (2) with easy access to instruments that facilitate the visualisation of nystagmus during the DHT and (3) the lack of training on how to perform these manoeuvres [17].

To improve this situation, a German protocol of a cluster-randomised trial was published in 2018 to evaluate the benefits of a half-day training for GPs [18]. A similar study protocol for ER physicians published in the USA in the same year [19] showed that physicians in the intervention group were twice more likely to perform the DHT and EM than doctors in the control group [20].

Our group has recently published a clinical trial [21] in patients with BPPV-PC that compared the performance of a single EM with the simulated manoeuvres performed by family physicians who had participated in a 2-h training workshop. This trial demonstrated that after receiving brief training, GPs can effectively diagnose and treat patients with BPPV-PC. However, the real challenge is to integrate these diagnostic and treatment manoeuvres into the everyday practice of all primary care physicians.

The objective of this study is to assess if a training intervention for GPs can optimise the diagnosis and management of patients with vertigo.

### Objectives {7}

#### Hypothesis

Better training on the diagnosis and treatment of vertigo and BPPV will improve the adherence of professionals to the vertigo clinical practice guidelines.

#### General objective

The general objective is to evaluate the effectiveness of a blended training intervention (online and face-to-face) regarding adherence to the BPPV clinical practice guidelines for the diagnosis and management of vertigo in PC.

#### Specific objectives

The following are the specific objectives:

- Assess if the diagnostic codes of vertigo in the primary care teams (PCT) that have received the training (intervention group (IG)) have improved compared to PCT in the control group (CG): decrease of nonspecific diagnoses such as dizziness and vertigo and increase in specific diagnoses such as BPPV, vestibular neuritis, Menière’s disease (see Supplementary material 1 for the complete list of diagnoses considered specific and nonspecific)
- Assess if referrals to ear, nose and throat (ENT) and neurology specialists due to vertigo are lower in the IG compared to the CG
- Assess if there is a decrease in the prescription of antivertigo agents in the IG compared to the CG
- Assess if the sick leave duration (in days) in the IG is shorter than in the CG

### Trial design {8}

To measure the effectiveness of the training in the IG, we designed a community multicentre cluster-randomised open-label trial, with the PCT as the allocation unit. The professionals of the PCT in the IG will receive training at the beginning of the study. PCT in the CG will be offered the training after study completion. Adherence to clinical practice guidelines will be measured during the year after completion of training in each PCT.

## Methods: participants, interventions and outcomes (Fig. 1)

### Study setting {9}

The study setting is Metropolitana Sud Area – Catalan Institute of Health (ICS), which in 2016 had 56 PCT for a catchment population of 1,334,381 people, of which 1,042,920 had been visited during that same year. A random sample of 20 PCT will be chosen from the 56; 10 will be included in the IG, and 10 in the CG.

**Fig. 1:**
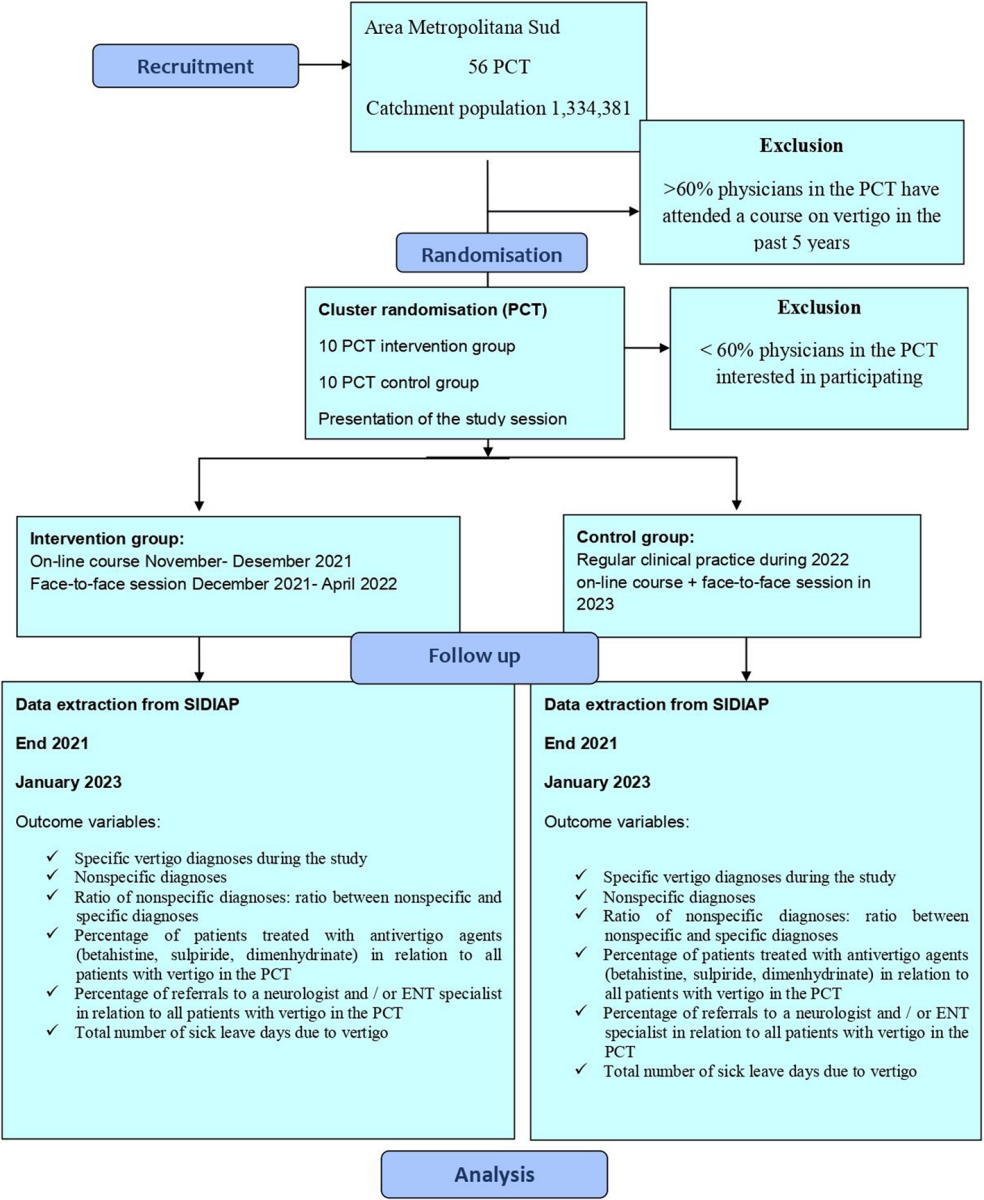
Study flowchart

The study population is all patients seen by family physicians (GPs) working in the selected PCT in the Area Metropolitana Sud of the ICS.

The study periods are pre-intervention study period from January to December 2021 and post-intervention period from January to December 2022.

### Eligibility criteria {10}

The inclusion criteria are all patients assigned to the 20 randomised primary care teams of the 56 that make up the Southern Metropolitan Area of the Catalan Health Institute, which serves a population of 1.3 million people.

The following are the exclusion criteria:

- Primary care teams in which > 60% of their professionals have received a course on vertigo in the last 5 years
- Primary care teams not interested in participating in the stud
- Patients who die during the year before and after the training intervention.
- Patients who change PCT

### Who will take informed consent? {26a}

The study will use a pseudonymised database in the Information System for the Development of Research in Primary Care (SIDIAP).

The study researchers will organise a session to explain and discuss the clinical trial with the various PCT. An information leaflet with the main aspects of the trial and the consent forms will be provided. After this session, the researchers will obtain the consent of the physicians who wish to participate.

### Additional consent provisions for collection and use of participant data and biological specimens {26b}

This study will not collect biological samples.

No new studies or sub-studies based on these data are anticipated.

### Interventions

#### Explanation for the choice of comparators {6b}

##### Active comparator: training group

The professionals of the primary care teams in the intervention group will receive the training at the beginning of the study.

##### Other: training on vertigo

The 14-h web-based course on vertigo management in primary care will include theory and clinical cases. The course will contain videos purposely produced for this course. Feedback (correct or incorrect) will be provided after each answer. Participants can ask questions during the course on the online platform. After the online course, researchers will deliver a 90-min face-to-face session where all participants will perform the diagnostic and therapeutic manoeuvres and where these manoeuvres will be evaluated and standardised.

##### No intervention: regular primary care practice

The professionals of the primary care teams that are in the control group will be offered training after the completion of the study.

#### Intervention description {11a}

VERTAP clinical trial (training intervention to improve the management of vertigo in primary care).

The objective of this protocol is to evaluate the efficacy of blended online and face-to-face training on the diagnosis and treatment of vertigo, in order to improve the adherence of family doctors to clinical practice guidelines.

Using the CAMFiC Moodle platform, we will adapt a pre-existing course prepared by the VERTAP that will contain videos showing the main diagnostic and therapeutic manoeuvres for vertigo. The contents will be reviewed by family doctors, physiotherapists and an ENT specialist. It will conform to the objectives of the study, with interactive clinical cases to improve the diagnosis and treatment of patients with vertigo. The online course requires approximately 14 h of individual study. This course will have to be completed within 6 weeks from the moment of registration and access.

After training with ENT and vestibular rehabilitation specialists, medical researchers will conduct the face-to-face session to verify and discuss the correct performance of the manoeuvres. The face-to-face group session lasts approximately 90 min. It will be carried out by a pair of researchers going to the PCT, who will supervise each doctor’s implementation of the diagnostic and therapeutic manoeuvres learned in the online course. During this session, researchers will also standardise the diagnosis and management of vertigo. Once the study period is over, the CG will be offered the possibility of taking the course.

An online course follow-up team will send email reminders regarding the start, follow-up and completion of the course. VERTAP researchers will take the register in the face-to-face sessions.

The TIDieR checklist will be followed during the intervention.

#### Criteria for discontinuing or modifying allocated interventions {11b}

No specific criteria have been proposed for discontinuing or modifying allocated interventions, since for the patient they do not differ from routine consultations.

#### Strategies to improve adherence to interventions {11c}

The PCT in Catalonia are currently focused on the COVID-19 pandemic. We will start the intervention when regular primary care activity resumes.

To increase adherence to the online course, we will send weekly reminders to encourage participants to complete the course.

To ensure participation in the face-to-face sessions, we will implement the training in the PCT of participating physicians. The director of the PCT will decide the best date for the face-to-face training within a 4-month period.

#### Relevant concomitant care permitted or prohibited during the trial {11d}

The PCT that have received previous training on vertigo have been excluded.

Only participants in the intervention group will be able to activate the online platform during the 6 weeks. After completion of the study, participants in the control group will be offered the online and face-to-face training.

#### Provisions for post-trial care {30}

No damages or compensation are anticipated for study participants. Physicians in the IG not able to complete/to pass the course will be able to retake it together with the CG.

### Outcomes {12}

Data from the whole population assigned to participating PCT will be obtained from the Information System for the Development of Research in PC (SIDIAP) at baseline and during a year after the start of the training.

#### Exposure variable

Measurement will be based on primary and secondary outcomes.

1. Primary outcome measures:
  - Register of ICD-10 vertigo diagnoses: Specific diagnoses registered during the study: BPPV (H81.1), Menière’s vertigo (H81) and vestibular neuritis (H81.2). Unspecific diagnoses registered during the study: dizziness or vertigo (R42) and other peripheral vertigos (H81.3). The ratio of specific diagnoses: ratio of specific versus nonspecific diagnoses. Effectiveness: higher proportion of specific diagnoses and lower of nonspecific diagnoses in the IG compared to the CG (see Supplementary material 1 for the complete list of diagnoses considered specific and nonspecific)
2. Secondary outcome measures:
  - Percentage of patients treated with antivertigo agents (betahistine, sulpiride, dimenhydrinate) in relation to all patients with vertigo in the PCT. Effectiveness: less antivertigo agents prescribed in the IG compared to the CG
  - Percentage of referrals to neurology and/or ENT due to vertigo (H81.1, H81.1, H81.3 and R42). Effectiveness: lower number of referrals in the IG compared to the CG
  - Total number of sick leave days due to vertigo in the IG. Effectiveness: less sick leave days in the IG compared to the CG

#### Participant timeline {13}

Work plan and chronogram (Table 1)

- Phase 1—Preparatory: meeting of all the researchers for the work protocol and the manual of procedures, planning of web-based course, elaborate the course materials and recording of the videos showing the diagnostic and therapeutic manoeuvres on simulated patients

**Table 1:** Schedule of enrollment, interventions and assessments

	***Study period***					
	Enrollment	Allocation	Post-allocation			Follow-ups
**Time point**^**a**^	t1		t2	t3	t4	t1–t4
**Enrollment**	x					x
**Eligibility screen**	x					x
**Presentation of the study**			x			x
**Allocation**	x					x
**Interventions**			x			x
**Online training**			x			x
**Face-to-face session**			x			x
**Assessments**				x		x
**Period of information collection**				x	x	x
**Analysis of results**					x	x
**Dissemination of the results**						x

^a^List specific time points: *t1* November 2019 to August 2021, *t2* September 2021 to March 2022, *t3* January 2022 to December 2022, *t4* January 2023 to December 2023

This phase will start in November 2019 and last until March 2021 (this period was extended to May 2021 due to the COVID 19 pandemic). The VERTAP team will meet with the ENT specialist once monthly.

The SIDIAP will randomise the participating centres and assist in defining the study variables.

- Phase 2: Presentation of the study to 20 PCT in the Southern Metropolitan Area of the Catalan Institute of Health from June to September 2021—participating PCT will be randomly assigned to the IG or the CG.

The IG will start the course at the beginning of the study, and PCT in the CG will start the training after the study follow-up period has ended.

The PCT in the IG will access the course online platform during November and December 2021. The face-to-face sessions will take place from January to April 2022.

- Phase 3: The period of information collection will be extended to January 2022 to December 2022.
- Phase 4: Analysis of results—data will be analysed by the researchers and a statistician. Manuscript writing will last from January to March 2023. The results will be published in scientific journals and disseminated in national and international conferences.

#### Sample size {14}

To improve vertigo diagnostic codes, accepting an alpha risk of 0.05 and a beta risk < 0.2 in a two-sided test, a total of 356 patients are needed per group (IG and CG) to detect as statistically significant a difference of 30% and 40% in correct diagnoses (BPPV, vestibular neuritis, vestibular migraine and Menière’s disease) for the CG and IG, respectively, with no loss to follow-up anticipated (GRANMO calculator, Institut Municipal d’Investigació Mèdica, Barcelona, Spain, using the ARCSINUS approach). To evaluate the final sample size, we take into account that the intercluster correlation coefficient (ICC) most commonly used in cluster-randomised trials in PC is 0.05. This ICC translated to a size of 45 individuals (assuming 10 clusters per group) in the design is adjusted by 1.24, i.e. *n* = 712 × 1.24 = 884 (45 per centre).

Similarly, the comparison of expected variation proportions for the prescription of antivertigo agents is 58.21% and 48% for the CG and IG, respectively (374 per group before adjustment by design) and 20% and 10% for referrals to neurology and ENT (195 per group before adjustment for design). All proportion estimates are based on the results of the previously published study [20].

However, data from *all* patients with a new diagnosis of vertigo and dizziness during the study period (1 year after the intervention) in participating centres will be collected, ensuring a number greater than the sample calculation.

#### Recruitment {15}

Recruitment will be carried out in the Primary Care Management of the Southern Metropolitan Area of the Catalan Institute of Health, which in 2016 had 53 PCT for a catchment population of 1,334,381 people, of whom 1,042,920 had been visited during that same year. A random sample of 20 PCT will be chosen from the 53; 10 will be included in the IG and 10 in the CG.

Each primary health centre will be personally visited to offer physicians the possibility of participating in the study.

### Assignment of interventions: allocation

- We have included all the “Metropolitana Sud” PCT.
- We have excluded centres that have taken BPPV courses and training.
- We have excluded the paediatric centres and PCT in prisons.
- From the remaining centres, a random function has selected 30 centres. A second randomisation has classified these PCT into two groups (control and intervention).

#### Sequence generation {16a}

From a list of 56 potential PCT, we will exclude the centres that have received training in vertigo in the last 5 years. Next, we will conduct two random processes using the RAND function in MySQL as follows: (1) a random selection of 30 of the remaining PCT (2) will be assigned to treatment groups (sampling without replacement a vector of 15 controls and 15 interventions).

#### Concealment mechanism {16b}

Randomisation will be applied to a list of PCT in the Southern Metropolitan Area of the Catalan Institute of Health. In consecutive order, we will inform the management teams of the centres and ask for their participation. We will hold informative sessions in the centres that accept participation and exclude the centres where intended participation is under 60% of professionals. The first eligible 10 PCT from each treatment group will define the final sample.

#### Implementation {16c}

The selection and assignment randomisations will be carried out by an external SIDIAP data manager. The researchers will contact the PCT managers to inform them. If managers agree to participate, a pair of researchers will attend the PCT and if 60% of the PCT professionals agree, they will be randomly assigned to the IG or the CG prior to the presentation session.

### Assignment of interventions: blinding

#### Who will be blinded {17a}

Not applicable due to the characteristics of the study, even the data analysis will be open-label.

#### Procedure for unblinding if needed {17b}

Not applicable due to the characteristics of the study.

### Data collection and management

#### Plans for assessment and collection of outcomes {18a}

The IG PCT will receive online and face-to-face training on the diagnosis and treatment of vertigo in general and of BPPV in particular, and also on the correct ICD-10 diagnostic codes. After the completion of the study, the CG PCT will be offered the same training. All data will be extracted from the SIDIAP pseudonymised database, a research platform that includes the computerised primary care medical records of 80% of the Catalan population.

Primary outcome: improvement in the specific and non-specific diagnoses of vertigo. Secondary outcome: comparison between the IG and CG of percentage of patients treated with antivertigo agents, and percentage of referrals to neurology and ENT.

#### Plans to promote participant retention and complete follow-up {18b}

After randomisation, the IG will make every reasonable effort to complete the study. The anticipated annual loss-to-follow-up is ≤ 5%. Physicians in the IG should commit to take the online and face-to-face course and apply the procedures learned during the study.

The PCT of Catalonia are currently focused on the COVID-19 pandemic. However, it is expected that PCT will resume normal primary care activities shortly. To increase adherence to the online course, participants will receive a weekly email reminder. To optimise participation in the face-to-face session in each PCT, we will contact the PCT director so that they decide the best date within a 4-month period. A pair of researchers will go to each PCT to deliver the face-to-face session.

#### Data management {19}

Clinical trial data will be entered electronically. Variables will be extracted from the SIDIAP before starting the training and quarterly during the 12 months after the start of the trial. The data of all patients with a new diagnosis of vertigo and dizziness in participating centres will also be collected, ensuring a sample size greater than necessary. The files will be safely stored in numerical order in one location for a period of 3 years after the end of the study. Only SIDIAP staff will be able to export these data.

#### Confidentiality {27}

The data will be anonymised, encrypted and collected by computerised extraction without the need to consult other data in the medical records. Only data of interest will be extracted.

The statistical code and datasets analysed during the current study will be available from the corresponding author on reasonable request.

#### Plans for collection, laboratory evaluation and storage of biological specimens for genetic or molecular analysis in this trial/future use {33}

Not applicable, this study does not involve the collection of biological samples. Not applicable due to the characteristics of the study.

## Statistical methods

### Statistical methods for primary and secondary outcomes {20a}

Data analyses will follow Consort Cluster recommendations (http://www.equator-network.org/reporting-guidelines/consort-cluster/), and intention-to-treat analysis will be used for comparisons between the groups. We will firstly analyse the baseline comparability of the two groups to verify homogeneity regarding study variables. Descriptive statistics will be performed for all baseline variables, using Student’s *t* or Mann-Whitney *U* test for quantitative variables, and chi-square or Fisher’s exact test for categorical variables.

The *t*-test will evaluate differences in outcome variables between the groups pre- and post-intervention. Logistic regression multivariate analysis will be performed to analyse the relationship between baseline variables of primary care centres and professionals, and outcome variables. Significance will be set at 5% for all analyses. Statistical software R (revised version 3.2.4) will be used.

### Interim analyses {21b}

Interim analyses and termination of the trial are not anticipated.

### Methods for additional analyses (e.g. subgroup analyses) {20b}

No additional analyses are anticipated.

### Methods in analysis to handle protocol non-adherence and any statistical methods to handle missing data {20c}

Data will be analysed according to the Consort Cluster guideline (http://www.equator-network.org/reporting-guidelines/consort-cluster/), and we will use intention-to-treat for comparisons between the groups. We will firstly analyse the baseline comparability of the two groups to verify homogeneity regarding study variables.

We propose to declare medical treatment as non-inferior to the intervention only if proven by intention-to-treat analysis.

### Plans to give access to the full protocol, participant-level data and statistical code {31c}

The statistical code and datasets analysed during the current study will be available from the corresponding author on reasonable request.

The trial registration can be found at: ClinicalTrials.gov Identifier: NCT04929444. Registered June 18, 2021, https://clinicaltrials.gov/ct2/show/NCT04929444

### Oversight and monitoring

The management structure comprises the principal investigator (IP), VERTAP members and a collaborating centre that will manage the data.

The VERTAP members are responsible for conducting the clinical trial. They will meet monthly to discuss the progress of the trial. During these meetings, the PI will verify that the study objectives are being met according to schedule.

The VERTAP constitutes the Steering Committee. They will periodically review the progress of the study.

The statistic analysis will be performed by the database research centre and the VERTAP statistician. Additional follow-up may be performed at the discretion of the monitoring manager.

### Composition of the coordinating centre and trial steering committee {5d}

#### Organisational structure

JEPP is the principal investigator and JLBM is in charge of organising the meetings.

All VERTAP members (JLBM, JEPP, YRM, RCM, IVB, SCM, VMR and EPR) participated in the design of the study. XGC is the ENT specialist, and ANC is the physiotherapist. These researchers are in charge of reviewing the study protocol.

IVB, YRM and EPR will prepare and follow up the web-based course, in collaboration with CAMFiC.

RCM, SCM and VMR will prepare and follow up the face-to-face sessions.

#### Trial steering committee

The Trial Steering Committee is exclusively composed of VERTAP researchers. The principal investigator will monitor the progress of the study and approve protocol changes.

The leader of the Steering Committee is JLBM. He is responsible for study planning, organising meetings, providing a report at each meeting and submitting reports to the Ethics Committee, managing the budget and contractual issues with individual centres, advising the researchers, determining the dates of presentation of the project to the centres and start of the interventions and the organisation of VERTAP.

#### Data managers

JAO and OCP are the data managers. The SIDIAP will perform the baseline statistical analysis. The CAMFiC will collaborate in the preparation of the web-based course.

All authors have contributed to improve the study protocol and have approved the final version of the manuscript.

### Composition of the data monitoring committee, its role and reporting structure {21a}

Since the original SIDIAP database is external to the study, and the data is obtained at two specific moments in time through computerised extraction; there is no role in this study for a data monitoring committee. The SIDIAP, JAO and OCP will be responsible for data extraction.

#### Adverse event reporting and harms {22}

During training, GPs were cautioned about the possibility that the way they talked about vertigo with patients could elicit a negative reaction.

#### Frequency and plans for auditing trial conduct {23}

The Trial Steering Committee will meet at least every 2 months to audit the development of the study, and the Ethics Committee will review the trial annually.

#### Plans for communicating important protocol amendments to relevant parties (e.g. trial participants, ethical committees) {25}

Any change or deviation in the protocol detected by the Trial Steering Committee will be communicated and evaluated by the Ethics Committee. Once approved, the PI will communicate it to the primary care teams. Any deviations from the protocol will be fully documented using a breach report form, and we will update the protocol in the clinical trial registry.

#### Dissemination plans {31a}

During 2023-2024, we will disseminate the results in national and international conferences and publish the results in journals of impact.

## Discussion

The study aims to evaluate the effect of a management training activity to increase GP adherence to vertigo clinical practice guidelines and consequently increase the rate of specific diagnoses such as BPPV, vestibular neuritis and Menière disease. Specific diagnoses, particularly of BPPV, should be followed by specific treatments such as repositioning manoeuvres, a decrease in referrals to other specialties (mainly ENT and neurology) and a decrease in sick leave days. In addition to BPPV, the most common cause of vertigo, other common causes of peripheral vertigo such as vestibular migraine are also underdiagnosed [22]. Similarly, the head impulse test for the diagnosis of vestibular neuritis is not routinely performed in PC consultations [23]. As a result, even in ENT clinics patients with vertigo mostly receive nonspecific diagnoses such as “dizziness” (67%) or “vestibular function disorder, unspecified” and are prescribed ineffective antivertigo agents, especially in cases of BPPV [24].

Geser and Straumann found that the number of BPPV diagnoses doubled with subsequent specialist evaluation and that nonspecific diagnoses decreased from 70 to 10% after a neuro-otological assessment, underscoring the need to improve neuro-otological skills among primary care physicians [25].

According to a comprehensive review on the economic burden of vertigo in Germany, 82% of patients with vertigo underwent magnetic resonance imaging (MRI) or computerised axial tomography (TAC) before being visited by a specialist for vestibular problems. In contrast, the head impulse test and the DHR were only performed in 5% and 34% of cases, respectively. Similarly, the best evidence-based treatment (the Epley repositioning manoeuvre) was performed in only 15% of patients with BPPV, while vertigo patients were prescribed an average of 1.8 medications. The mean sick leave for patients with vertigo during a 12-month observation period was 69 days, with an estimated cost of USD 12,542.

**Fig. 2:**
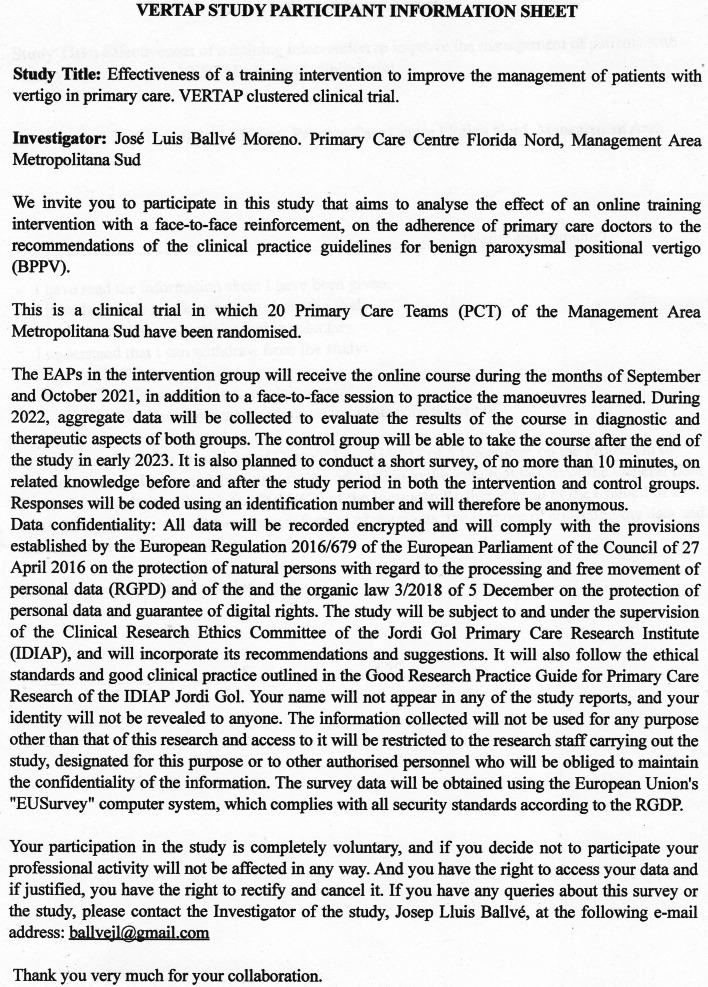
VERTAP study participant information sheet

**Fig. 3:**
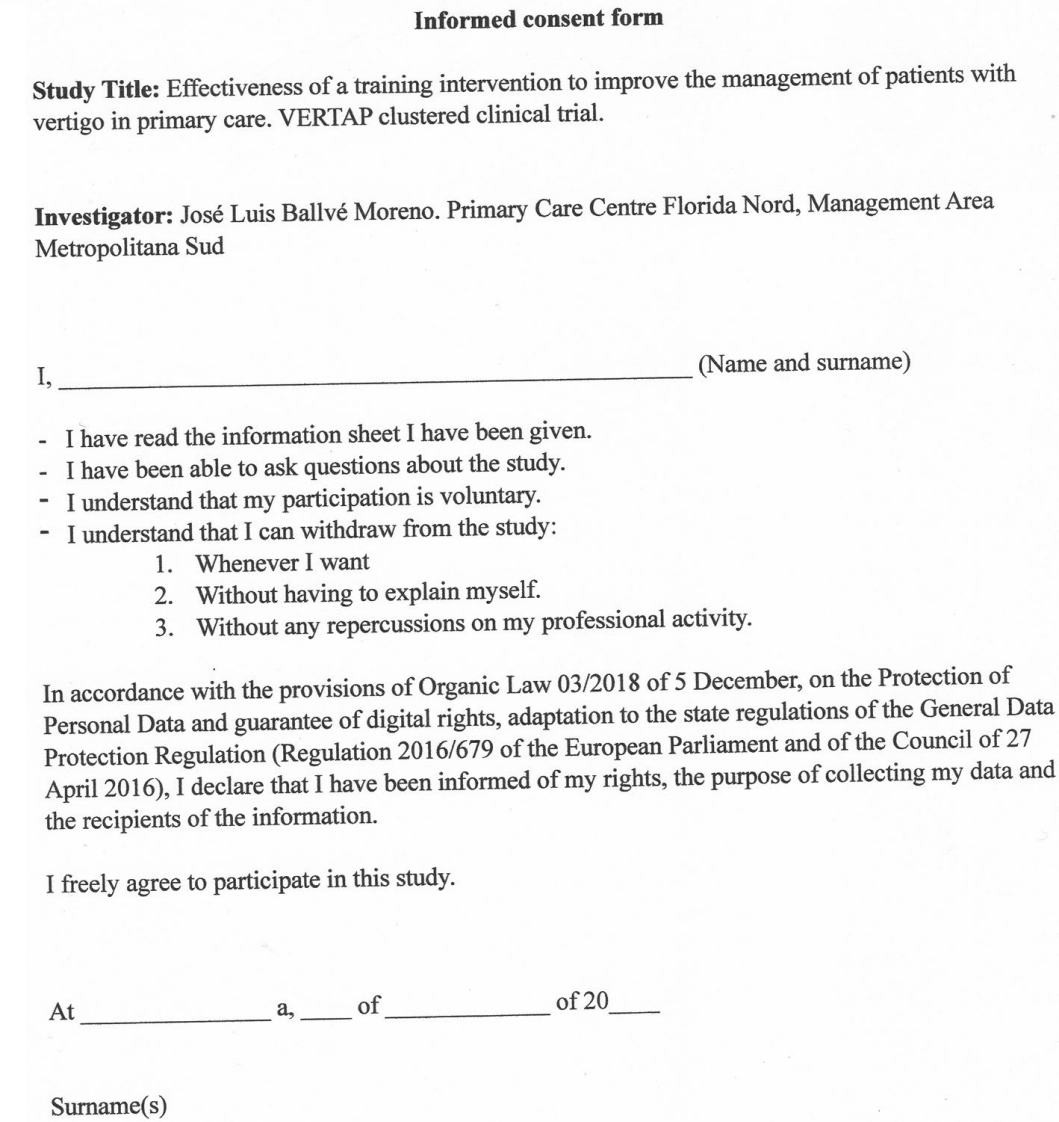
Informed consent

In summary, we are faced with an underuse of inexpensive diagnostic tests that require specific training, and an overuse of mostly unnecessary, low diagnostic performance, expensive medical tests. Finally, medications that do not improve the condition and might even be contraindicated are excessively prescribed. In contrast, inexpensive and simple treatments that would result in a rapid improvement of many of these patients (especially patients with BPPV) but that require training of professionals remain largely underused [26], Figs. 2 and 3.

## Trial status

The trial is recruiting participants since October 2021. The completion date is scheduled for the end of December 2021. All PCT completed the course in December 2021, although some face-to-face visits were delayed until April 2022 due to the pandemic.

Protocol version 1.0.

The following image presents the VertAP trial logo.

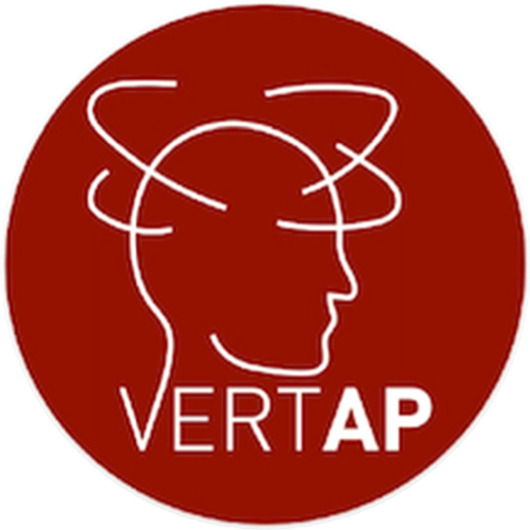



## Supplementary Information

**Additional file 1.**

## Data Availability

The dataset will be available in a public repository after the acceptance of the manuscripts. Data can be provided upon reasonable request.

## Abbreviations

BPPV: Benign paroxysmal positional vertigo
PC: Primary care
GP: General practitioner, family doctor
CAMFiC: Catalan Society of Family and Community Medicine
CG: Control group
IG: Intervention group
ENT: Ear, nose and throat
BPPV-PC: Benign paroxysmal positional vertigo of the posterior canal
DHT: Dix Hallpike test
EM: Epley manoeuvre
PCT: Primary care team
ICS: Catalan Institute of Health
TD: Temporary disability
ICC: Intercluster correlation coefficient
SIDIAP: Information System for the Development of Research in Primary Care
IDIAP: Primary Care Research Institute
LSSI: Information Society and Email Services
