# Correction: Effectiveness of a training intervention to improve the management of vertigo in primary care: a multicentre cluster-randomised trial, VERTAP

**DOI:** 10.1186/s13063-022-06759-y

**Published:** 2022-09-21

**Authors:** Jenniffer Elizabeth Pérez Patiño, José Lluís Ballvé Moreno, Yolanda Rando Matos, Jesús Almeda Ortega, Oriol Cunillera Puértolas, Ricard Carrillo Muñoz, Iván Villar Balboa, Xavier González Compta, Olga Lucía Arias Agudelo, Sebastiá Calero Muñoz, Vanessa Monforte Rodríguez, Anna Navarro Cortes, Eva Peguero Rodríguez

**Affiliations:** 1 Primary Care Centre Sant Martí de Provençals, Management Area of Barcelona, Catalan Institute of Health, Barcelona, Spain; 2 Vertigo Approach Research Group in Primary Care (VERTAP), Fundació Institut Universitari per la Recerca en Atenció Primària de Salut Jordi Gol i Gurina (IDIAPJGol), Barcelona, Spain; 3 Primary Care Centre Florida Nord, Management Area Metropolitana Sud, Catalan Institute of Health, Barcelona, Spain; 4 University of Barcelona, Barcelona, Spain; 5 Research Support Unit Metropolitana Sud, Institut Universitari d’Investigació en Atenció Primària Jordi Gol (IDIAP Jordi Gol), Cornellà de Llobregat, Spain; 6 Primary Care Centre Florida Sud. Management Area Metropolitana Sud, Catalan Institute of Health, Barcelona, Spain; 7 Ear, Nose and Throat, Department, University Hospital Bellvitge, Barcelona, Spain; 8 Management Area Metropolitana Sud, Catalan Institute of Health, Barcelona, Spain; 9 Primary Care Centre, Management Area Metropolitana Sud, Catalan Institute of Health, Barcelona, Spain; 10 Rehabilitation Centre Viladecans, Management Area Metropolitana Sud, Catalan Institute of Health, Barcelona, Spain; 11 Primary Care Centre Castelldefels, Management Area Metropolitana Sud, Catalan Institute of Health, Barcelona, Spain

**Correction: Trials 23, 208 (2022)**

**https://doi.org/10.1186/s13063-022-06548-7**

Following the publication of the original article [1], the authors have requested that we add the below text to the Funding section:

*The project has received a research grant from the Instituto de Salud Carlos III, of the Ministry of Economy and Competitiveness (Spain), awarded in the 2019 call within the Action of the Health Strategy 2013-2016, within the National Programme of Research oriented to the Challenges of Society, within the National Plan for Technical, Scientific and Innovation Research 2013-2016, with reference PI19/00736, co-financed with ERDF funds from the European Union (European Regional Development Fund).*

The original article has been corrected.
