# Supplementary material for: Effectiveness of a training intervention to improve the management of vertigo in primary care: a multicentre cluster-randomised trial, VERTAP
Source: Trials. 2022 Jul 29;23:608. doi: 10.1186/s13063-022-06548-7 (PMC9335455; doi:10.1186/s13063-022-06548-7)
Supplement: Supplementary file 1 — Additional file 1. [file 13063_2022_6548_MOESM1_ESM.docx]

DIAGNOSES:

SPECIFIC DIAGNOSES- Benign Paroxysmal Positional Vertigo (H81.10, H81.11,12,13), Meniere's vertigo (H81.0,01, 02, 03 and 09), vestibular neuronitis (H81.20,21,22 and 23 ) and Central vertigo (H81.4, H81.41 to 43 I 49) Labyrinthitis H83.01 Labyrinthitis, right ear H83.02 Labyrinthitis, left ear H83.03 Labyrinthitis, bilateral H83.09 Labyrinthitis, ear unspecified H83.1 Fistula labyrinthine H83.11 Labyrinthine fistula, right ear H83.12 Labyrinthine fistula, left ear H83.13 Labyrinthine fistula, bilateral H83.19 Labyrinthine fistula, unspecified ear H83.2 Labyrinthine dysfunction Labyrinthine hypersensitivity Labyrinthine hypofunction Loss of lossX labyrinthine H83.2X1 Labyrinthine dysfunction, right ear H83.2X2 Labyrinthine dysfunction, left ear H83.2X3 Labyrinthine dysfunction, bilateral H83.2X9 Labyrinthine dysfunction, ear unspecified.

NON-SPECIFIC DIAGNOSIS NON-SPECIFIC: dizziness or vertigo (R42) or AURAL vertigo (H81.31, H81.311,312, 313 I 319, H81.39) or unspecified disorder of vestibular function (H81.9, H81 .90 to 93, H81 .8X1, H818X2, H81.8X3 and H81.8X9, h82) Epidemic vertigo (H88.1), Infrared vertigo (T75.23XA, T75.23XD, T75.23XS), Other types of Peripheral vertigo (H81.391,392,393 and 399, H81.4), dizzying syndromes in Diseases classified elsewhere (82.1,82.2,82.3,82.9, H83, H83.0) diagnosed during the study period.
